# Editorial: Metabolic estimates during glucose challenge tests and continuous glucose monitoring—Innovative and broad approaches to assessing glucose and insulin metabolism in diverse populations

**DOI:** 10.3389/fphys.2022.1112502

**Published:** 2022-12-16

**Authors:** Joon Ha, Melanie Cree-Green, Stephanie Therese Chung, Cecilia Diniz Behn

**Affiliations:** ^1^ Department of Mathematics, Howard University, Washington, DC, United States; ^2^ Department of Pediatrics, University of Colorado Anschutz Medical Campus, Aurora, CO, United States; ^3^ National Institute of Diabetes and Digestive and Kidney Diseases (NIH), Bethesda, MD, United States; ^4^ Department of Applied Mathematics and Statistics, Colorado School of Mines, Golden, CO, United States

**Keywords:** oral glucose tolerance test (OGTT), continuous glucose monitoring, beta-cell function, insulin sensitivity, glucose fluctuation

## Introduction

Identifying early signs of metabolic dysfunction is crucial for preventing and delaying type 2 diabetes (T2D). As such, glucose challenge tests that assess fasting and postprandial glucose and related hormonal factors (e.g., insulin/C-peptide, glucagon) provide critical information on pathophysiological mechanisms of type 2 diabetes (T2D). Glucose challenge tests range in complexity from intravenous glucose tolerance tests (IVGTT) that are technically challenging and require specialized metabolic testing centers to mixed meal and oral glucose tolerance tests (OGTT) that can be conducted in outpatient settings. While existing simple mathematical indices (e.g., insulinogenic index (IGI), oral disposition index, HOMA-IR, and Matsuda index [1, 2] are widely used in clinical and epidemiological studies, more complex mathematical models of glucose challenge tests are emerging as sensitive and precise markers for beta-cell function and insulin sensitivity [3, 15, 16]. Recently, continuous glucose monitoring (CGM)-derived metabolic parameters, including mean amplitude of glycemic excursions (MAGE), offer pragmatic alternatives to assess metabolic risk and status [4].

The development of new measures for quantifying data from glucose challenge tests and CGM is necessary to support clinical practice and promote scientific understanding. This Research Topic sought to highlight new and emerging modeling approaches and markers based on data from glucose challenge tests and CGM with the potential for advancing the field of diabetes risk prediction and assessment especially in diverse populations. Given the variability in metabolic responses associated with age, sex, and race/ethnicity, mathematical models and diagnostic markers must be tested in a wide range of patient populations [10]. Moreover, the application of these approaches in varied populations often requires re-examination of the assumptions and application of mathematical models and diagnostic markers. In particular, several articles address the promising possibilities raised by analysis of CGM data. We provide an overview of the published articles below.

### Novel modeling approaches

Minimal models have been widely used in mathematical modeling of metabolism [15]. However, in cases where more data are available, models that incorporate additional metabolites provide insights into specific aspects of metabolic dysregulation. Several articles in this Research Topic proposed novel mathematical models of the interacting dynamics of different metabolites. Subramanian et al. introduced a model of coupled glucose-insulin-glucagon dynamics during an isoglycemic intravenous glucose infusion (IIGI) experiment designed to mimic an OGTT. This model was used to identify several differences between participants with T2D relative to weight matched control participants without diabetes. Abohtyra et al. described a model-based method for inferring a parameterization of insulin secretion rate using glucose, insulin, and C-peptide data from an OGTT even when sampling of these data was sparse. Hampton et al. presented a mathematical model of glycerol-insulin dynamics that considered how the dynamics of glycerol suppression and recovery probe the function of adipose tissue and its response to insulin in adolescent girls. They found that the dynamics of glycerol differ from the dynamics of glucose in this population, thereby emphasizing the need to consider age/life-stage in metabolic assessments.

### Markers for improving diabetes screening and treatment

There is much effort focused on improving T2D screening and understanding diabetes progression. Several papers in this Research Topic addressed this question while considering the modifying factors of demographics and genetics. Shi et al. investigated the prevalence and significance of low muscle mass and its relationship to glycemia. Low muscle mass was associated with glycemic excursions in males but not females. Richter et al. used a data assimilation approach to predict glycemic states in adolescents following bariatric surgery. They first estimated parameters in a mechanistic model using data assimilation on clinical OGTT data [11]; then they applied logistic regression models with variables including these parameters as well as clinical data from the electronic health record to predict post-surgical glycemic control. Vejrazkova et al. analyzed OGTT data and found that the G allele of the rs10830963 polymorphism is associated with impaired early phase of beta cell function. Interestingly, this impairment was present even in healthy individuals with normoglycemia. Karamched et al. described the concept of delay-induced uncertainty (DIU) and the implications of DIU for glucose fluctuations. They established that DIU was present in large regions of parameter space for an established model of glucose-insulin dynamics [13, 14], and they argued that DIU is pathogenic for obesity and type 2 diabetes. These theoretical models are important as they explore diagnostic screening tools as well as mechanisms for diabetes progressions. Additional experimental data are needed to evaluate these provocative model predictions.

## Markers of glucose fluctuations

There has been much effort to find markers of glucose excursions [5, 6, 7, 12] to identify patients who are at high risk for progression to diabetes and its complications [8, 9]. In-line with one of the goals of this Research Topic, novel metabolic markers or model parameters of glucose challenge tests, the following two articles found novel markers of glucose fluctuations. Ha et al. showed that the discrepancy between glucose management indicator and HbA1c is a good predictor for intensive care unit (ICU) stay and mortality. Jagannathan et al **.** Identified that elevated 1-h glucose at the time of remission of T2D dysglycemia is a risk factor for T2D relapse among Black patients with obesity. However, large fluctuations in blood glucose concentrations are not the only indicators for high risk for worsened glycemia and diabetic complications: glucose variability may also play a role. New methods leveraging CGM hold much promise for the screening and monitoring of diabetes with a focus on glucose variability. Wang et al. used CGM data to relate glucose variability to risk for nocturnal hypoglycemia in patients with T2D. Similarly, in another article, Wang et al. used CGM data to compare the effects of basal insulin *vs.* premixed insulin on glucose variability and hypoglycemia in T2D patients. However, Faerch et al. found that there was poor agreement between measurements from venous blood plasma and CGM during an OGTT. More work is needed to understand the relationship between CGM data and typical plasma-based measures of glucose dynamics.

## Conclusion and future directions

In conclusion, the fields of method and model development to understand glucose and related hormone dynamics are active, with new emerging ideas. The articles in this Research Topic highlighted novel modeling approaches and markers based on data from glucose challenge tests and CGM that could improve T2D risk prediction, screening, and glucose control. The primary need for the future will be to determine how to translate the research based-methods presented here into simpler models with broadly clinically relevant endpoints. This will require additional studies of glucose metabolism in the postprandial state. Ideally, this would allow for more sophisticated risk assessments of dysglycemia and clinical methods for assessing beta-cell function relative to insulin sensitivity. Such methods could be translated to large epidemiologic studies and move us closer to clinical precision medicine.

